# The Effect of Season and Meteorological Conditions on Parasite Infection in Farm-Maintained Mouflons (*Ovis aries Musimon*)

**DOI:** 10.1155/2022/1165782

**Published:** 2022-01-25

**Authors:** B. Pilarczyk, A. Tomza-Marciniak, R. Pilarczyk, N. Sadowska, J. Udała, J. Kuba

**Affiliations:** ^1^Department of Animal Reproduction Biotechnology and Environmental Hygiene, West Pomeranian University of Technology Szczecin, ul. Klemensa Janickiego 29, 71-270 Szczecin, Poland; ^2^Laboratory of Biostatistics, West Pomeranian University of Technology Szczecin, ul. Klemensa Janickiego 29, 71-270 Szczecin, Poland

## Abstract

Due to their limited environment, farm animals are at greater risk of parasitic infection than free-living animals, which also have greater natural resistance to parasitic diseases. The aim of the present study was to determine the influence of season and meteorological conditions (temperature and humidity) on the species composition and dynamics of parasitic infections in farmed mouflons. The study was conducted in a herd of mouflon (*n* = 40) in an extensive system: the animals remained on pasture all year round. The herd was dewormed twice per year with albendazole. Fecal samples were collected at monthly intervals over three years and tested. The prevalence of infection was defined based on coproscopic methods. For most of the studied protozoans (except for *E. parva*), a greater prevalence was recorded in spring and summer (i.w. from May to September). Regarding nematodes, *Capillaria* spp., *Nematodirus* sp., and the Trichostrongylidae demonstrated a much greater prevalence in winter (i.e., in January and December). Temperature and precipitation were found to be positively correlated with intensity of infection by protozoans. However, maximum air temperature was negatively correlated with infection intensity by some nematodes. The deworming practice used in the herd (selection of substance, date, and method of dosing) did not effectively protect the mouflons against parasitoses. Changes in the microclimate resulted in high extent and intensity of mouflon infection with gastrointestinal parasites. Understanding the dynamics of parasitic infections in mouflons during the year allows the development of an appropriate preventive programme.

## 1. Introduction

The European mouflon (*Ovis aries musimon* Schreber, 1782) is a wild mountain sheep naturally occurring in Asia. The species first arrived in Poland at the beginning of the 20^th^ century, mainly for the purpose of enriching the selection of game animals. However, the mouflon was not well suited to the Polish climate and is nowadays kept under farmed or semicaptive conditions [1].

Even if kept under farm conditions, mouflons (*Ovis aries musimon*) are very vulnerable to different diseases, including parasitic infections; these negatively affect the welfare of the animals and the flock by causing in emaciation and weakness, or possibly even death [2, 3]. The most significant economic impacts are the indirect losses resulting from poor feed conversion, low body weight gain, reduced milk yield of nursing mothers, metabolic disorders, reduced immunity of animals, and the need for additional veterinarian care [4]. Such diseases clearly have a serious impact on animal's health and the profitability of mouflon breeding.

Free-living animals have some natural resistance to parasitic diseases. In addition, some naturally growing plant species, such as garlic (*Allium sativum*), wormwood (*Artemisia absinthium*), black walnut (*Juglans nigra*), mugwort (*Artemisia vulgaris*), or thyme (*Thymus vulgaris*) also show antiparasitic properties [5–7]. Furthermore, animals kept in a restricted environment, like a farm, are at much greater risk of parasitic infection [8].

The prevalence of parasitic infection is governed by a range of factors, including breeding conditions, grazing system, and seasonality of microclimatic conditions. Additionally, a key role in the spread of parasitic infections is played by the state of pastures, because the eggs excreted with faeces hatch as larvae; under appropriate conditions (temperature and humidity), they can reach the invasive stage. Understanding the dynamics of parasitic infections in mouflons during the year allows the development of an appropriate preventive programme [9, 10]. As such, monitoring parasitic infections occurring on mouflon farms is an essential part of herd management.

The aim of our present study was to determine the influence of season and meteorological conditions (temperature and humidity) on the species composition and dynamics of parasitic infections in farmed mouflons.

## 2. Materials and Methods

### 2.1. Animals

The coproscopy studies were performed on a farm located at the edge of Puszcza Notecka in the Lubuskie voivodeship, where mouflons are kept for hobby and educational purposes. The herd of mouflons (*n* = 40) was kept in an extensive system where they remained on pasture all year round. The grazing system was determined by locating the animals in pastures, with the animals staying in each pasture for approximately six weeks. Throughout the year, the animals had unlimited access to freshwater and salt licks (NaCl: >95%, H_2_O: <0.5%, and components insoluble in water: <3%). In summer, pasture fodder was the primary source of feed. In addition, the animals received meadow hay and straw. In winter, however, in addition to straw and hay, the animals were fed carrots or fodder beets, crushed oats, corn, and sugar beet pomace. The animals also received a mineral-vitamin mixture.

The herd was dewormed twice per year with albendazole (10 g/100 ml). Healthcare for the herd was provided by the owners, and the farm workers are directly responsible for the animals. The use of veterinary services was ad hoc, usually in response to animal health problems. There were no natural water reservoirs (artificial waterholes) in the pastures. The mouflons themselves were imported from the Czech Republic.

### 2.2. Meteorological Data

Data on precipitation and air temperature were obtained from the measurement and observation station of the Institute of Meteorology and Water Management-National Research Institute, located 14 km from the farm. The temperature and precipitation measurements taken during the three-year research period are given in Supplementary Table 1.

### 2.3. Parasitological Tests

In total, 426 faecal samples were collected at monthly intervals during three years of sampling. The prevalence (proportion of host individuals infected with a particular parasite, %, [11]) and intensity of infection (number of individuals of a particular parasite species in a single host, [11]) were defined based on coproscopic studies performed with the Willis-Schlaf and McMaster methods [12]. The species composition of coccidia was established with Pellerdi's key [13]. These tests were supplemented with oocyst cultures performed in a moist chamber at 24-26°C. A 2.5%-aqueous solution of potassium dichromate (K_2_Cr_2_O^7^) was used as a mould-prevention agent. The species composition of the gastrointestinal nematodes was determined based on cultures of larvae hatched from isolated eggs, according to the recommendations of Gundłach and Sadzikowski [14]. Fluke eggs were detected by the decantation method [15].

### 2.4. Statistical Analysis

The results were analysed with Statistica 13.3 (TIBCO Software Inc., Palo Alto, USA). The *χ*^2^ test was used to analyse the effect of season (year) on the prevalence, whereas the significant differences in parasite infections between the sampling seasons (years) were checked with the nonparametric Kruskal-Wallis test. The correlation between climate variables and intensity of infection was determined by Spearman's rank-order correlation, because the intensity of infection (number of parasites per gram of faeces) did not demonstrate a normal distribution for either protozoans or nematodes. The level of statistical significance was assumed at *P* ≤ 0.05.

## 3. Results

The prevalence of *Eimeria* protozoans and gastrointestinal nematodes in mouflons in experimental period is presented in Table 1. Differences in prevalence were observed between different years in *Eimeria bakuensis* (*χ*^2^ = 17.8; *P* ≤ 0.001), *E. ovinoidalis* (*χ*^2^ = 8.22; *P* ≤ 0.02), *Strongyloides* spp. (*χ*^2^ = 34.65; *P* ≤ 0.001), and Trichostrongylidae (*χ*^2^ = 31.03; *P* ≤ 0.001). These resulted from a significantly higher prevalence of infections observed in the third year of the experiment. The mean infection intensity of *Eimeria intricata*, *Strongyloides* sp., *Trichuris ovis*, *Nematodirus* sp., *Trichostrongylidae*, and *Chabertia ovina* was significantly (*P* ≤ 0.05) higher in the first year of the experiment (Table 2).

For most of the studied protozoans (except for *E. parva*), an effect of season on the prevalence of infection was observed. A much higher prevalence of infection was found in the spring and summer seasons (Table 3). For gastrointestinal nematodes, the effect of season on the prevalence of infection was found only in *Capillaria* sp. and *Nematodirus* sp. A significantly greater prevalence of infection was noted in winter than in the other seasons (Table 3). Regarding infection intensity, all *Eimeria* protozoans demonstrated significantly (*P* ≤ 0.05) higher mean intensity in spring and summer, while the nematodes were found to have significantly greater total mean infection intensity (*P* ≤ 0.05) in winter than in spring (Table 4).

**Figure 1:**
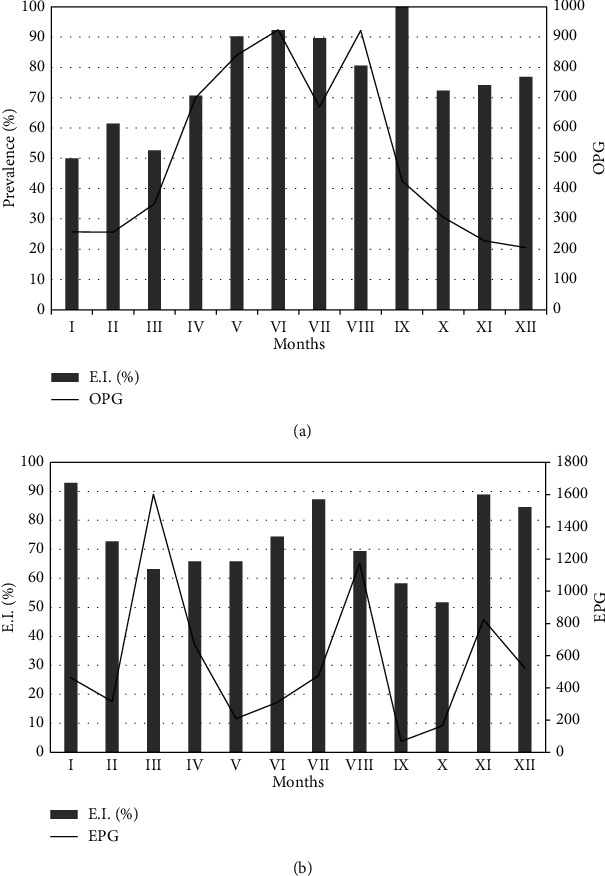
Prevalence and intensity of infection of mouflons with *Eimeria* protozoans (a) and gastrointestinal nematodes (b) in annual cycle.

For most of the studied protozoans (except for *E. parva*), month affected the prevalence of their infection. A much greater prevalence was recorded from May to September. For nematodes, an effect of month on the prevalence was found for *Capillaria* sp., *Nematodirus* sp., and *Trichostrongylidae*. A much greater prevalence was recorded in January and December and in July and August than in the other months (Table 5, Figure 1).

The average infection intensity of *Eimeria* protozoans in May, June, and August was significantly (*P* ≤ 0.05) higher than that in January, February, March, November, and December and higher in April and July than in February, November, and December (Figure 1, Supplementary Table S2 and S3).

The correlation analysis showed that the intensity of protozoan infection was significantly and positively correlated with temperature (*r* = 0.43, *P* ≤ 0.001) (Table 6). This correlation was found to be weak for *E. parva* and *E. ovinoidalis*, (*r* = 0.25 and *r* = 0.20, *P* ≤ 0.01; respectively), but average for *E. bakuensis* and *E. crandalis* (*r* = 0.36, *P* ≤ 0.001). For the *Chabertia ovina* nematodes, a significant negative correlation (*r* = −0.21, *P* ≤ 0.05) was found between the intensity of infection and the maximum temperature. The intensity of protozoan infection was also significantly positively correlated with precipitation; however, this correlation was weak (*r* = 0.17, *P* ≤ 0.0.01).

## 4. Discussion

Continuous parasitological monitoring has become an indispensable element of animal husbandry in recent years. Systematic control of parasites as well as the use of prevention programmes developed individually for a particular farm is now an obligatory activity. During the period of parasitological monitoring, the prevalence and intensity of mouflon infection with gastrointestinal parasites were found to vary with the seasons.

Mature individuals not showing clinical signs but infected with a small number of parasites may constitute themselves as healthy hosts and reservoirs of parasitic stages for the environment. In natural habitats, mouflons often change their location and feeding grounds, resulting in “natural cleansing” of the environment from invasive forms of parasites, as they simply die without being able to find a host. In turn, animals kept in closed breeding facilities always remain in the same area, causing an accumulation of invasive parasites. Cabaret et al. [9] found a significantly higher prevalence (%) and mean intensity of coccidial infection (OPG) in captive mouflons (73.17% and 814.6 OPG, respectively) than in wild mouflons (36.73% and 112.7 OPG). In our study, the mean prevalence of infection of mouflons was found to be quite high: 75.91% with *Eimeria* protozoans and 72.93% with gastrointestinal nematodes. In addition, our findings also indicate an increase in extent over subsequent years (Table 1), which may be explained by the gathering of mouflons in a limited area and the accumulation of oocysts/eggs in the environment. Our findings are also higher than the prevalence of coccidial infection identified in previous studies. Ferraro et al. [16] showed a lower prevalence of coccidial infection in mouflons in Italy (53.33%) than that in our study. Similar results were also obtained by Verin et al. [17] in mouflons from Apuane Alps Park (58.2%) and by Magi et al. [18] in mouflons living in Monti Livornesi Park (67%). In addition, the prevalence of infection in free-ranging mouflons from Lower Silesia (Poland) was found to be 58.27% for gastrointestinal nematodes and 44.6% for coccidia [2].

In most cases, mouflons experience multiple infections with coccidia. Correlation analysis showed that the intensity of protozoan infection was significantly and positively correlated with the ambient temperature. This may be related with the dispersal strategy of the parasite. Oocysts are excreted into the environment with the faeces, but are not yet invasive. Only under the effect of favourable environmental conditions, such as the presence of oxygen and sufficient temperature and humidity, do oocysts turn infective (sporocysts). Infective sporulated oocysts show high resistance to unfavourable conditions in the external environment (they can survive for several months). The factors that favour the survival of coccidial oocysts are high humidity and favourable temperatures. A low temperature inhibits the sporogony phase and lowers the ability of sporulation under optimal conditions. Studies have found that only 10% of the oocysts reached the sporoblast stage after 14 days of storage at 4°C, and most of the remaining unsporulated oocysts degenerated, even after placing them in an area with a favourable temperature (30°C) [11, 19–21]. In our study, a significant increase in the prevalence of infection with *Eimeria* protozoans was observed from May to September and in the intensity of infection from May to August (Figure 1), which may indicate the weather conditions favourable for the development of parasites at that time. However, the impact of local climate on external parasite stages should be confirmed with further research. Sheep are closely related to mouflons, and thus, the species of coccidia typical in sheep are also found in mouflons. Antoszek and Balicka-Ramisz [22] showed the highest prevalence of infection in sheep from July to September. This shift in the prevalence and intensity of infection may well be due to climate change (warming).

In pastures, infected mouflons shed faeces, increasing the risk of parasite infection. For gastrointestinal nematodes, the process of larval development occurs in the external environment and depends primarily on air temperature, soil humidity, and oxygen access. Under favourable climatic conditions, the risk of contamination increases rapidly [16, 23, 24].

At higher temperatures in summer, larvae move quickly and hence consume more nutrients. When there are no longer nutrients, the larvae stop moving and die. This process is accelerated by water loss, which evaporates more quickly from the environment and from the body of larvae during warm periods. In colder seasons, the larvae are not very mobile, curl into a spiral, and are able to survive for a long period of time [23, 24].

In the present study, a significant negative correlation was found between the intensity of infection and maximum environmental temperature for *Chabertia ovina* nematodes. Paciejewski [23] suggests that drought inhibits the hatching of larvae in some gastrointestinal nematodes, which may result in a lower intensity of invasion. Under favourable conditions (20-25°C) and high air humidity, the larvae leave their eggshells after 16-30 days. Other authors mention that the development of larvae depends mainly on temperature.

In our study, a significant increase in the prevalence of infection with gastrointestinal nematodes was observed in January, July and October. In addition, the intensity of infection peaked in March and again in August (Figure 1). Undoubtedly, climatic conditions play a crucial role in spreading infection. The presence of infective eggs depends on weather conditions. The high prevalence of mouflon infection with gastrointestinal nematodes was noted in winter (January), suggesting that favourable microclimatic conditions occurred in that period. In addition, the risk of contamination may have been greatly increased by the concentration of many animals in a small area and the use of a pasture breeding system. Winter feeding could have been one of the reasons for the high extent of infection with gastrointestinal nematodes in January. The animals were fed with additional food: hay was put into the feeders, and the animals gathered around them. This feeding strategy required many individuals to gather together in one place, with a high concentration of excrement, trampling the ground that was often muddy. This could well have caused the observed increased prevalence of infection.

## 5. Conclusion

In general, the prophylactic procedures adopted on the farm (selection of substance, date and method of dosing) did not effectively protect the mouflons against parasitoses, as indicated by the high extent of infection (>70%). It was also found that the extent of infection was influenced by the season: nematodes were more prevalent in winter and *Eimeria* in the spring and summer seasons.

Microclimatic changes resulted in a high prevalence and intensity of mouflon infection with gastrointestinal parasites. Understanding the dynamics of parasitic infections in mouflons during the year can allow the development of an efficient strategic deworming programme.

## Figures and Tables

**Table 1:** Prevalence (%) of *Eimeria* protozoans and gastrointestinal nematodes in mouflons in the experimental period.

Parasites	Year I		Year II		Year III		*χ* ^2^	*P* value	Total	
	*n*_*i*_/*n*	E.I. (%)	*n*_*i*_/*n*	E.I. (%)	*n*_*i*_/*n*	E.I. (%)			*n*_*i*_/*n*	E.I. (%)
*Eimeria parva*	45/175	25.7	42/152	27.6	34/99	34.3	2.38	*P* = 0.30	121/426	28.4
*E. bakuensis* (syn. E. ovina)	69/175	39.4	64/152	42.1	64/99	64.6	17.80	*P* ≤ 0.001	197/426	46.2
*E. crandalis*	48/175	27.4	36/152	23.7	25/99	25.3	0.60	*P* = 0.74	109/426	25.6
*E. intricata*	42/175	24.0	34/152	22.4	20/99	20.2	0.53	*P* = 0.77	96/426	22.5
*E. ovinoidalis*	70/175	40.0	66/152	43.4	57/99	57.6	8.22	*P* = 0.02	193/426	45.3
Total protozoans	134/175	76.0	109/152	71.7	77/99	77.8	1.37	*P* = 0.50	319/426	74.9
*Strongyloides* sp.	60/175	34.3	65/152	42.8	70/99	70.7	34.65	*P* ≤ 0.001	195/426	45.8
*Capillaria* sp.	42/175	24.0	24/152	15.8	24/99	24.2	4.04	*P* = 0.13	90/426	21.18
*Trichuris ovis*	30/175	17.1	23/152	15.1	21/99	21.2	1.55	*P* = 0.46	74/426	17.4
*Nematodirus* sp.	59/175	33.7	27/152	17.8	20/99	20.2	12.58	*P* = 0.002	106/426	24.9
*Chabertia ovina*	42/175	24.6	25/152	16.4	27/99	27.3	4.95	*P* = 0.08	95/426	22.3
Trichostrongylidae	71/175	40.6	70/152	46.1	74/99	74.7	31.03	*P* ≤ 0.001	215/426	50.5
Total nematodes	122/175	69.7	110/152	72.4	80/99	80.8	4.06	*P* = 0.13	312/426	73.2

*n*_*i*_/*n*: number of infected animals/number of tested animals.

**Table 2:** Intensity of infection with protozoans (oocysts per gram (OPG)) and gastrointestinal nematodes (eggs per gram (EPG)) in mouflons.

Parasites	Year I			Year II			Year III			Kruskal-Wallis test	Total		
	Mean	Me	Range	Mean	Me	Range	Mean	Me	Range		Mean	Me	Range
*Eimeria* protozoans													
*E. parva*	99	50	50-500	112	50	50-500	112	100	50-200	*H* = 4.4; *P* = 0.11	107	50	50-500
*E. bakuensis* (syn. *E. ovina*)	347	150	50-1200	367	200	50-2500	273	150	50-1200	*H* = 2.2; *P* = 0.34	329	200	50-2500
*E. crandalis*	155	100	50-500	204	200	50-800	152	100	50-600	*H* = 4.2; *P* = 0.12	171	100	50-800
*E. intricata*	120^a^	100	50-300	78^b^	50	50-400	185^a^	100	50-800	*H* = 12.2; *P* = 0.002	119	100	50-800
*E. ovinoidalis*	399	250	50-1400	354	250	50-2200	396	400	50-1100	*H* = 1.5; *P* = 0.47	383	300	50-2200
Total protozoans	513^a^	275	50-2950	560^a^	300	50-5100	658^b^	575	50-2400	*H* = 10.5; *P* = 0.005	564	300	50-5100
Gastrointestinal nematodes													
*Strongyloides* sp.	323^a^	225	50-1300	200^b^	100	50-1500	195^b^	100	50-1000	*H* = 7.8; *P* = 0.02	236	100	50-1500
*Capillaria* sp.	199	100	50-800	102	50	50-450	113	50	50-500	*H* = 5.7; *P* = 0.06	150	100	50-800
*Trichuris ovis*	272^a^	150	50-800	111^ab^	100	50-450	67^b^	50	50-100	*H* = 12.3; *P* = 0.002	164	100	50-800
*Nematodirus* sp.	264^a^	100	50-2300	115^b^	100	50-600	95^b^	100	50-300	*H* = 8.4; *P* = 0.02	194	100	50-2300
*Chabertia ovina*	290^a^	300	50-800	116^b^	50	50-600	81^b^	50	50-300	*H* = 19.9; *P* ≤ 0.001	185	100	50-800
Trichostrongylidae	437^a^	400	50-1200	189^b^	100b	50-1200	374^a^	100	50-2600	*H* = 23.6; *P* ≤ 0.001	334	100	50-2600
Total nematodes	785^a^	600	50-3600	335^b^	200	50-2600	590^a^	300	50-3350	*H* = 45.3; *P* ≤ 0.001	575	300	50-3600

**Table 3:** Prevalence (%) of *Eimeria* protozoans and gastrointestinal nematodes in mouflons in specific seasons.

Parasites	Winter		Spring		Summer		Autumn		*χ* ^2^	*P*	Total	
	*n*_*i*_/*n*	E.I. (%)	*n*_*i*_/*n*	E.I. (%)	*n*_*i*_/*n*	E.I. (%)	*n*_*i*_/*n*	E.I. (%)				
*Eimeria* protozoans												
*E. parva*	28/124	22.6	34/121	28.1	38/99	38.4	21/82	25.6	7.24	*P* = 0.06	121/426	28.4
*E. bakuensis* (syn*. E. ovina*)	46/124	37.1	74/121	61.2	52/99	52.5	25/82	30.5	24.76	*P* ≤ 0.001	197/426	46.2
*E. crandalis*	15/124	12.1	39/121	32.2	42/99	42.4	13/82	15.9	33.48	*P* ≤ 0.001	109/426	25.6
*E. intricata*	10/124	8.1	30/121	24.8	30/99	30.3	26/82	31.7	22.6	*P* ≤ 0.001	96/426	22.5
*E. ovinoidalis*	35/124	28.2	75/121	62.0	58/99	58.6	25/82	30.5	42.49	*P* ≤ 0.001	193/426	45.3
Total protozoans	68/124	54.8	102/121	84.3	88/99	88.9	61/82	74.4	42.42	*P* ≤ 0.001	319/426	74.9
Gastrointestinal nematodes												
*Strongyloides* sp.	67/124	54.0	49/121	40.5	45/99	45.5	34/82	41.5	5.38	*P* = 0.14	195/426	45.8
*Capillaria* sp.	38/124	30.6	20/121	16.5	18/99	18.2	14/82	17.1	9.60	*P* = 0.02	90/426	21.18
*Trichuris ovis*	18/124	14.5	20/121	16.5	19/99	19.2	17/82	20.7	1.64	*P* = 0.65	74/426	17.4
*Nematodirus* sp.	48/124	38.7	17/121	14.0	23/99	23.2	18/82	22.0	20.8	*P* ≤ 0.001	106/426	24.9
*Chabertia ovina*	34/124	27.4	25/121	20.7	20/99	20.2	16/82	19.5	2.68	*P* = 0.44	95/426	22.3
Trichostrongylidae	59/124	47.6	54/121	44.6	55/99	55.6	47/82	57.3	4.52	*P* = 0.21	215/426	50.5
Total nematodes	95/124	76.6	83/121	68.6	73/99	73.7	61/82	74.4	2.12	*P* = 0.55	312/426	73.2

*n*_*i*_/*n*: number of infected animals/number of tested animals.

**Table 4:** Intensity of infection with *Eimeria* protozoans (oocysts per gram (OPG)) and gastrointestinal nematodes (eggs per gram (EPG)) in mouflons in specific seasons.

Parasites	Winter			Spring			Summer			Autumn			Kruskal-Wallis test
	Mean	Me	Range	Mean	Me	Range	Mean	Me	Range	Mean	Me	Range	
*Eimeria* protozoans													
*E. parva*	71^a^	50	50-200	132^b^	100	50-500	128^ab^	100	50-500	76^a^	50	50-200	*H* = 17.0; *P* < 0.001
*E. bakuensis* (syn. *E. ovina*)	164^a^	100	50-800	431^b^	375	50-1200	410^b^	250	50-2500	166^a^	100	50-600	*H* = 26.8; *P* < 0.001
*E. crandalis*	120^ab^	50	50-400	213	200^a^	50-600	181^a^	100	50-800	69^b^	50	50-200	*H* = 17.0; *P* < 0.001
*E. intricata*	105	100	50-250	162	100	50-800	107	50	50-300	88	50	50-400	*H* = 3.6; *P* = 0.30
*E. ovinoidalis*	197^a^	100	50-600	490^b^	450	50-1200	416^bc^	300	50-2200	244^ac^	200	50-600	*H* = 23.5; *P* < 0.001
Total protozoans	284^a^	150	50-1450	830^b^	750	50-2450	686^c^	400	50-5100	247^a^	200	50-1050	*H* = 66.9; *P* < 0.001
Gastrointestinal nematodes													
*Strongyloides* sp.	251	150	50-1200	180	100	50-1300	293	150	50-1500	212	100	50-850	*H* = 4.4; *P* = 0.21
*Capillaria* sp.	129	100	50-500	138	50	50-550	192	100	50-800	171	75	50-800	*H* = 1.0; *P* = 0.81
*Trichuris ovis*	169	100	50-800	108	100	50-300	197	50	50-750	185	100	50-750	*H* = 0.1; *P* = 0.99
*Nematodirus* sp.	191	100	50-650	265	50	50-2300	174	100	50-600	164	100	50-700	*H* = 1.7; *P* = 0.64
*Chabertia ovina*	199	100	50-600	142	50	50-800	183	75	50-600	225	100	50-600	*H* = 4.7; *P* = 0.19
Trichostrongylidae	443	100	50-2600	243	100	50-1200	335	250	50-1200	303	100	50-1200	*H* = 2.2; *P* = 0.54
Total nematodes	696^a^	425	50-3350	401^b^	200	50-3600	636^ab^	350	50-2600	559^ab^	250	50-2700	*H* = 14.0; *P* = 0.003

^a,b^Different lowercase letters indicate statistically significant differences at *P* ≤ 0.05.

**(a):** 

Month	*Eimeria* protozoans											
	*E. parva*		*E. bakuensis*		*E. crandalis*		*E. intricata*		*E. ovinoidalis*		Total protozoans	
	*n*_*i*_/*n*	E.I. (%)	*n*_*i*_/*n*	E.I. (%)	*n*_*i*_/*n*	E.I. (%)	*n*_*i*_/*n*	E.I. (%)	*n*_*i*_/*n*	E.I. (%)	*n*_*i*_/*n*	E.I. (%)
January	6/42	14.3	13/42	31.0	8/42	19.0	5/42	11.9	9/42	21.4	21/42	50.0
February	10/44	22.7	19/44	43.2	3/44	6.8	1/44	2.3	16/44	36.4	27/44	61.4
March	12/38	31.6	14/38	36.8	4/38	10.5	4/38	10.5	10/38	26.3	20/38	52.6
April	10/41	24.4	20/41	48.8	9/41	22.0	8/41	19.5	16/41	39.0	29/41	70.7
May	11/41	26.8	29/41	70.7	14/41	34.1	8/41	19.5	26/41	63.4	37/41	90.2
June	13/39	33.3	25/39	64.1	16/39	41.0	14/39	35.9	33/39	84.6	36/39	92.3
July	15/39	38.5	22/39	56.4	18/39	46.2	14/39	35.9	30/39	76.9	35/39	89.7
August	15/36	41.7	16/36	44.4	15/36	41.7	7/36	19.4	18/36	50.0	29/36	80.6
September	8/24	33.3	14/24	58.3	9/24	37.5	9/24	37.5	10/24	41.7	24/24	100.0
October	7/29	24.1	8/29	27.6	5/29	17.2	8/29	27.6	11/29	37.9	21/29	72.4
November	8/27	29.6	6/27	22.2	3/27	11.1	10/27	37.0	7/27	25.9	20/27	74.1
December	6/26	23.1	11/26	42.3	5/26	19.2	8/26	30.8	7/26	26.9	20/26	76.9
	*χ* ^2^ = 11.83; *P* = 0.38		*χ* ^2^ = 34.04; *P* ≤ 0.001		*χ* ^2^ = 40.28; *P* ≤ 0.001		*χ* ^2^ = 32.58; *P* ≤ 0.001		*χ* ^2^ = 71.47; *P* ≤ 0.001		*χ* ^2^ = 53.33; *P* ≤ 0.001	

**(b):** 

Month	Gastrointestinal nematodes													
	*Strongyloides* sp.		*Capillaria* sp.		*Trichuris ovis*		*Nematodirus* sp.		*Chabertia ovina*		*Trichostrongylidae*		Total nematodes	
	*n*_*i*_/*n*	E.I. (%)	*n*_*i*_/*n*	E.I. (%)	*n*_*i*_/*n*	E.I. (%)	*n*_*i*_/*n*	E.I. (%)	*n*_*i*_/*n*	E.I. (%)	*n*_*i*_/*n*	E.I. (%)	*n*_*i*_/*n*	E.I. (%)
January	24/42	57.1	19/42	45.2	7/42	16.7	24/42	57.1	14/42	33.3	21/42	50.0	39/42	92.9
February	23/44	52.3	9/44	20.5	4/44	9.1	13/44	29.5	10/44	22.7	20/44	45.5	32/44	72.7
March	20/38	52.6	10/38	26.3	7/38	18.4	11/38	28.9	10/38	26.3	18/38	47.4	24/38	63.2
April	16/41	39.0	8/41	19.5	6/41	14.6	7/41	17.1	8/41	19.5	18/41	43.9	27/41	65.9
May	14/41	34.1	7/41	17.1	5/41	12.2	6/41	14.6	8/41	19.5	17/41	41.5	27/41	65.9
June	19/39	48.7	5/39	12.8	9/39	23.1	4/39	10.3	9/39	23.1	19/39	48.7	29/39	74.4
July	23/39	59.0	8/39	20.5	11/39	28.2	11/39	28.2	10/39	25.6	27/39	69.2	34/39	87.2
August	15/36	41.7	9/36	25.0	7/36	19.4	11/36	30.6	10/36	27.8	19/36	52.8	25/36	69.4
September	7/24	29.2	1/24	4.2	1/24	4.2	1/24	4.2	0/24	0.0	9/24	37.5	14/24	58.3
October	7/29	24.1	4/29	13.8	4/29	13.8	3/29	10.3	3/29	10.3	10/29	34.5	15/29	51.7
November	15/27	55.6	3/27	11.1	7/27	25.9	8/27	29.6	5/27	18.5	18/27	66.7	24/27	88.9
December	12/26	46.2	7/26	26.9	6/26	23.1	7/26	26.9	8/26	30.8	19/26	73.1	22/26	84.6
	*χ* ^2^ = 18.94; *P* = 0.06		*χ* ^2^ = 24.92; *P* = 0.009		*χ* ^2^ = 12.45; *P* = 0.33		*χ* ^2^ = 42.36; *P* ≤ 0.001		*χ* ^2^ = 15.14; *P* = 0.17		*χ* ^2^ = 20.88; *P* = 0.03		*χ* ^2^ = 31.32; *P* ≤ 0.001	

**Table 6:** The results of the correlation analysis (Spearman's rank-order test) between the intensity of infection and climatic parameters.

Parasites	*N*	Mean temp.	Minimal temp.	Maximal temp.	Precipitation
		*r* value			
*E. parva*	121	0.25^∗∗^	0.24^∗∗^	0.25^∗∗^	0.19^∗^
*E. bakuensis* (syn. E. ovina)	197	0.36^∗∗∗^	0.33^∗∗∗^	0.39^∗∗∗^	0.14^∗^
*E. crandalis*	109	0.36^∗∗∗^	0.25^∗∗^	0.36^∗∗∗^	0.27^∗∗^
*E. intricata*	96	ns	ns	ns	ns
*E. ovinoidalis*	193	0.20^∗∗^	0.19^∗∗^	0.24^∗∗∗^	0.16^∗^
Total protozoans	322	0.43^∗∗∗^	0.39^∗∗∗^	0.45^∗∗∗^	0.17^∗∗^
*Strongyloides* sp.	195	ns	ns	ns	ns
*Capillaria* sp.	90	ns	ns	ns	ns
*Trichuris ovis*	74	ns	ns	ns	ns
*Nematodirus* sp.	106	ns	ns	ns	ns
*Chabertia ovina*	95	ns	ns	-0.21^∗^	ns
Trichostrongylidae	215	ns	ns	ns	ns
Total nematodes	316	ns	ns	ns	ns

∗*P* ≤ 0.05, ∗∗*P* ≤ 0.01, and ∗∗∗*P* ≤ 0.001; ns: nonsignificant (*P* > 0.05).

## Data Availability

The data used to support the findings of this study are available from the corresponding author upon request.

## Supplementary Materials

Supplementary Materials Supplementary table 1: range of temperatures (°C) and precipitation (mm) in experimental period. Supplementary table 2: intensity of infection (OPG) of mouflons with *Eimeria* protozoans in an annual cycle. Supplementary table 3: intensity of infection (EPG) of mouflons with gastrointestinal nematodes in an annual cycle.
