# Supplementary material for: The Effect of Season and Meteorological Conditions on Parasite Infection in Farm-Maintained Mouflons (Ovis aries Musimon)
Source: J Parasitol Res. 2022 Jan 25;2022:1165782. doi: 10.1155/2022/1165782 (PMC8808193; doi:10.1155/2022/1165782)
Supplement: Supplementary Materials — Supplementary table 1: range of temperatures (°C) and precipitation (mm) in experimental period. Supplementary table 2: intensity of infection (OPG) of mouflons with Eimeria protozoans in an annual cycle. Supplementary table 3: intensity of infection (EPG) of mouflons with gastrointestinal nematodes in an annual cycle. [file 1165782.f1.docx]

Supplementary table 1. Range of temperatures (°C) and precipitation (mm) in experimental period

| **Month** | **Temperature (I^st^ year)** | | | **Precipitation** | **Temperature (II^nd^ year)** | | | **Precipitation** | **Temperature (III^rd^ year)** | | | **Precipitation** |
| --- | --- | --- | --- | --- | --- | --- | --- | --- | --- | --- | --- | --- |
|  | **mean** | **min.** | **max.** |  | **mean** | **min.** | **max.** |  | **mean** | **min.** | **max.** |  |
| January | 3 | -6 | 10 | 94.8 | 1 | -6 | 11 | 71.9 | 2 | -10 | 10 | 43.1 |
| February | 0 | -11 | 10 | 48.9 | 2 | -2 | 8 | 46.6 | 8 | 3 | 11 | 15.2 |
| March | 10 | 2 | 18 | 15 | 3 | -3 | 11 | 31 | 13 | 7 | 20 | 33.1 |
| April | 15 | 5 | 28 | 37.1 | 13 | 2 | 25 | 17.1 | 18 | 9 | 23 | 55 |
| May | 20 | 13 | 28 | 36.8 | 20 | 12 | 27 | 115.2 | 20 | 11 | 31 | 96.3 |
| June | 21 | 14 | 29 | 121.5 | 24 | 14 | 36 | 109.2 | 23 | 18 | 34 | 49.6 |
| July | 24 | 18 | 30 | 154.4 | 27 | 19 | 37 | 32.6 | 28 | 22 | 34 | 61.4 |
| August | 24 | 20 | 33 | 96.4 | 27 | 21 | 36 | 44.9 | 24 | 19 | 34 | 104.3 |
| September | 20 | 19 | 29 | 25.7 | 19 | 13 | 25 | 45.2 | 21 | 14 | 27 | 92.6 |
| October | 13 | 4 | 20 | 43.4 | 15 | 11 | 20 | 22.2 | 16 | 9 | 24 | 50 |
| November | 8 | 3 | 12 | 63.1 | 7 | 2 | 13 | 46.4 | 9 | -1 | 15 | 12.1 |
| December | 1 | -4 | 10 | 37 | 5 | 2 | 8 | 26.7 | 4 | -2 | 11 | 82.8 |

Supplementary table 2. Intensity of infection (OPG) of mouflons with *Eimeria* protozoans in an annual cycle

| **Month** |  | ***E. parva*** | ***E. bakuensis* (syn*. E. ovina)*** | ***E. crandalis*** | ***E. intricata*** | ***E. ovinoidalis*** | **Total protozoans** |
| --- | --- | --- | --- | --- | --- | --- | --- |
| January | n | 6 | 13 | 8 | 5 | 9 | 21 |
|  | mean | 100^ab^ | 115^a^ | 131^ab^ | 110 | 189^ab^ | 257^ac^ |
|  | median | 50 | 100 | 100 | 100 | 100 | 150 |
|  | range | 50-200 | 50-300 | 50-300 | 100-150 | 50-500 | 50-750 |
| February | n | 10 | 19 | 3 | 1 | 16 | 27 |
|  | mean | 65 ^a^ | 171^a^ | 50^b^ | 50 | 175^a^ | 256^a^ |
|  | median | 50 | 100 | 50 | 50 | 75 | 100 |
|  | range | 50-100 | 50-800 | 50-50 | 50-50 | 50-600 | 50-1200 |
| March | n | 12 | 14 | 4 | 4 | 10 | 20 |
|  | mean | 63^a^ | 200^a^ | 150^ab^ | 113 | 240^ab^ | 350^ac^ |
|  | median | 50 | 125 | 75 | 75 | 175 | 150 |
|  | range | 50-100 | 50-500 | 50-400 | 50-250 | 50-500 | 50-1450 |
| April | n | 10 | 20 | 9 | 8 | 16 | 30 |
|  | mean | 185^b^ | 378^ab^ | 200^ab^ | 250 | 488^ab^ | 700^c^ |
|  | median | 125 | 300 | 100 | 225 | 450 | 625 |
|  | range | 100-500 | 50-1050 | 50-600 | 100-500 | 50-1100 | 50-2000 |
| May | n | 11 | 29 | 14 | 8 | 26 | 37 |
|  | mean | 127 ^ab^ | 443^ab^ | 225^a^ | 213 | 462^ab^ | 841^bc^ |
|  | median | 100 | 500 | 200 | 75 | 400 | 850 |
|  | range | 50-300 | 50-1200 | 50-600 | 50-800 | 50-1000 | 100-2400 |
| June | n | 13 | 25 | 16 | 14 | 33 | 37 |
|  | mean | 96 ^ab^ | 460^ab^ | 209^a^ | 82 | 514^b^ | 924^bc^ |
|  | median | 100 | 300 | 200 | 50 | 500 | 950 |
|  | range | 50-200 | 50-1200 | 50-450 | 50-250 | 50-1200 | 150-2450 |
| July | n | 15 | 22 | 18 | 14 | 30 | 36 |
|  | mean | 97 ^ab^ | 334^ab^ | 189^ab^ | 79 | 360^ab^ | 669^c^ |
|  | median | 50 | 200 | 150 | 50 | 300 | 550 |
|  | range | 50-200 | 50-1200 | 50-500 | 50-200 | 50-1300 | 50-2950 |
| August | n | 15 | 16 | 15 | 7 | 18 | 29 |
|  | mean | 180 ^ab^ | 659^b^ | 233^a^ | 143 | 500^b^ | 922^bc^ |
|  | median | 100 | 575 | 200 | 150 | 275 | 400 |
|  | range | 50-500 | 50-2500 | 50-800 | 50-300 | 50-2200 | 50-5100 |
| September | n | 8 | 14 | 9 | 9 | 10 | 24 |
|  | mean | 88 ^ab^ | 243^a^ | 78^ab^ | 122 | 430^ab^ | 425^abc^ |
|  | median | 50 | 225 | 100 | 50 | 350 | 275 |
|  | range | 50-250 | 50-500 | 50-500 | 50-250 | 50-850 | 50-1550 |
| October | n | 7 | 8 | 5 | 8 | 11 | 21 |
|  | mean | 64 ^a^ | 175^a^ | 100^ab^ | 138 | 268^ab^ | 305^abc^ |
|  | median | 50 | 150 | 50 | 100 | 300 | 300 |
|  | range | 50-150 | 50-350 | 50-200 | 50-400 | 100-400 | 50-1050 |
| November | n | 8 | 6 | 3 | 10 | 7 | 20 |
|  | mean | 81 ^a^ | 233^a^ | 50^b^ | 70 | 236^ab^ | 228^a^ |
|  | median | 50 | 100 | 50 | 50 | 200 | 150 |
|  | range | 50-200 | 50-600 | 50-50 | 50-100 | 50-600 | 50-700 |
| December | n | 6 | 11 | 5 | 8 | 7 | 20 |
|  | mean | 83 ^a^ | 123^a^ | 50^b^ | 63 | 214^ab^ | 205^a^ |
|  | median | 50 | 100 | 50 | 50 | 200 | 150 |
|  | range | 50-200 | 50-400 | 50-50 | 50-100 | 50-600 | 50-750 |

^a,b^ - different lowercase letters indicate statistically significant differences at *P* ≤ 0.05

Supplementary table 3. Intensity of infection (EPG) of mouflons with gastrointestinal nematodes in an annual cycle

| **Month** | | ***Strongyloides* sp.** | ***Capillaria* sp.** | ***Trichuris ovis*** | ***Nematodirus* sp.** | ***Chabertia ovina*** | ***Trichostrongylidae*** | **Total nematodes** |
| --- | --- | --- | --- | --- | --- | --- | --- | --- |
| January | n | 24 | 19 | 7 | 24 | 14 | 21 | 39 |
|  | mean | 179 | 118 | 79 | 210 | 189 | 152 | 462 |
|  | median | 125 | 100 | 50 | 100 | 100 | 100 | 350 |
|  | range | 50-600 | 50-500 | 50-150 | 50-650 | 50-550 | 50-400 | 50-1450 |
| February | n | 23 | 9 | 4 | 13 | 10 | 20 | 33 |
|  | mean | 111 | 56 | 88 | 165 | 140 | 173 | 315 |
|  | median | 100 | 50 | 75 | 100 | 50 | 100 | 250 |
|  | range | 50-300 | 50-100 | 50-150 | 50-400 | 50-550 | 50-800 | 50-950 |
| March | n | 20 | 10 | 7 | 11 | 10 | 18 | 24 |
|  | mean | 500 | 215 | 307 | 177 | 270 | 1083 | 1602 |
|  | median | 500 | 125 | 100 | 100 | 200 | 800 | 1625 |
|  | range | 50-1200 | 50-500 | 50-800 | 50-650 | 50-600 | 50-2600 | 100-3350 |
| April | n | 16 | 8 | 6 | 7 | 8 | 18 | 30 |
|  | mean | 272 | 238 | 142 | 543 | 325 | 361 | 667 |
|  | median | 50 | 125 | 100 | 150 | 300 | 100 | 150 |
|  | range | 50-1300 | 50-550 | 50-300 | 50-2300 | 50-800 | 50-1200 | 50-3600 |
| May | n | 14 | 7 | 5 | 6 | 8 | 17 | 28 |
|  | mean | 129 | 79 | 70 | 58 | 56 | 138 | 209 |
|  | median | 100 | 50 | 50 | 50 | 50 | 100 | 200 |
|  | range | 50-450 | 50-150 | 50-100 | 50-100 | 50-100 | 50-500 | 50-600 |
| June | n | 19 | 5 | 9 | 4 | 9 | 19 | 29 |
|  | mean | 139 | 60 | 106 | 88 | 56 | 224 | 310 |
|  | median | 100 | 50 | 100 | 100 | 50 | 150 | 250 |
|  | range | 50-300 | 50-100 | 50-200 | 50-100 | 50-100 | 50-800 | 50-950 |
| July | n | 23 | 8 | 11 | 11 | 10 | 27 | 34 |
|  | mean | 209 | 69 | 59 | 100 | 70 | 311 | 476 |
|  | median | 150 | 50 | 50 | 50 | 50 | 250 | 350 |
|  | range | 50-500 | 50-100 | 50-100 | 50-300 | 50-150 | 50-900 | 100-1350 |
| August | n | 15 | 9 | 7 | 11 | 10 | 19 | 25 |
|  | mean | 537 | 317 | 436 | 259 | 295 | 503 | 1172 |
|  | median | 400 | 200 | 450 | 200 | 300 | 500 | 1200 |
|  | range | 50-1500 | 50-800 | 100-750 | 50-600 | 50-600 | 50-1200 | 50-2600 |
| September | n | 7 | 1 | 1 | 1 | 0 | 9 | 14 |
|  | mean | 50 | 50 | 50 | 50 | -- | 50 | 68 |
|  | median | 50 | 50 | 50 | 50 | -- | 50 | 50 |
|  | range | 50-50 | 50-50 | 50-50 | 50-50 | -- | 50-50 | 50-100 |
| October | n | 7 | 4 | 4 | 3 | 3 | 10 | 14 |
|  | mean | 79 | 88 | 63 | 50 | 83 | 75 | 164 |
|  | median | 100 | 75 | 50 | 50 | 100 | 75 | 175 |
|  | range | 50-100 | 50-150 | 50-100 | 50-50 | 50-100 | 50-100 | 50-350 |
| November | n | 15 | 3 | 7 | 8 | 5 | 18 | 24 |
|  | mean | 320 | 517 | 314 | 244 | 400 | 403 | 823 |
|  | median | 200 | 700 | 100 | 100 | 450 | 300 | 425 |
|  | range | 50-850 | 50-800 | 50-750 | 50-700 | 50-600 | 50-1200 | 100-2700 |
| December | n | 12 | 7 | 6 | 7 | 8 | 19 | 22 |
|  | mean | 154 | 71 | 117 | 121 | 169 | 329 | 523 |
|  | median | 100 | 50 | 100 | 100 | 100 | 100 | 425 |
|  | range | 50-500 | 50-100 | 50-300 | 50-300 | 50-600 | 50-1200 | 50-1300 |
